# Nontypeable *Haemophilus influenzae* Otitis Media: Mastoiditis and Meningitis Complicated with Central Venous Thrombosis in an Immunocompetent Child

**DOI:** 10.1155/2021/8845200

**Published:** 2021-03-13

**Authors:** Erdem Gönüllü, Nesrin Özkan, Ahmet Soysal, Engin Acıoğlu, Emine Betül Tavil, Selin Nar Ötgün, Metin Karaböcüoğlu

**Affiliations:** ^1^Ataşehir Memorial Hospital, Clinic of Pediatrics, Istanbul, Turkey; ^2^Ataşehir Memorial Hospital, Clinic of Ear-Nose-Throat, Istanbul, Turkey; ^3^Microbiology Reference Laboratories Biological Products Directorate, General Directorate of Public Health, Ankara, Turkey

## Abstract

Implementation of the *Haemophilus influenzae* type B (Hib) conjugate vaccine brought about a reduction in the number of cases and morbidity from type B but an increase in nontypeable strain infections. Nontypeable *Haemophilus influenzae* (NTHi) commonly colonizes children's upper respiratory tract and causes otitis media, sinusitis, and bronchitis. Invasive NTHi diseases, such as meningitis and septicemia, have rarely been reported. Herein, we discuss a previously healthy, fully immunized 3-year-old girl presented with otitis media and mastoiditis leading to meningitis caused by NTHi complicated with central venous thrombosis. She was treated with antibiotics, mastoidectomy and ventilation tube insertion, and anticoagulation therapy and recovered uneventfully. Through this case, we wish to share our unique clinical experience that NTHi should be born in mind as a potential pathogen that can cause meningitis in previously healthy children, which may be helpful in future cases.

## 1. Introduction

*Haemophilus influenzae* is a Gram-negative coccobacillus that includes encapsulated (typeable) and unencapsulated (nontypeable) strains. Six encapsulated serotypes have been identified, designated as types a–f. Nontypeable strains do not have a polysaccharide capsule [1].

Before the global introduction *of H. influenzae* type B (Hib) immunization, 95% of invasive *H. influenzae* disease was caused by Hib [1, 2]. After the worldwide introduction of Hib immunization, invasive Hib disease incidence has markedly declined, and nontypeable *H. influenzae* (NTHi) has become a significant cause of invasive *H. influenzae* disease in vaccinated children [3].

The major clinical manifestations of NTHi infections in otherwise healthy children are otitis media (OM), sinusitis, conjunctivitis, and pneumonia [4]. Invasive infections, such as bacteremia and meningitis, rarely occur in newborns or immunocompromised hosts.

Here, we report a case of a previously healthy 3-year-old girl who developed meningitis and cavernous sinus thrombosis as a complication of acute otitis media and mastoiditis. We wish to emphasize that NTHi must be considered a potential pathogen that can cause invasive disease, even in previously healthy children.

## 2. Case Report and Methods

A previously healthy 3-year-old Turkish girl was admitted to our pediatric emergency unit with fever, a running nose, and ear pain, with no past medical history of note. She was fully immunized, including four doses of Hib and 13-valent pneumococcal conjugate vaccines. At present, her body temperature (tympanic) was 38°C, and her physical examination revealed purulent postnasal discharge, bilateral hyperemic and bulging tympanic membranes, normal lung and cardiac sounds, comfortable abdomen, and regular neurological evaluation. She was diagnosed with acute OM, and cefdinir was given orally for seven days.

Two weeks after the end of oral antibiotic treatment, the patient presented to our hospital again with fever complaints, a tendency to sleep, and purulent discharge from the right ear. Her physical examination showed a toxic-sleepy child with purulent discharge from the right ear, without neck stiffness or a Kernig's sign. A full septic screen was performed revealing a C-reactive protein level of 310 mg/L (normal range <11), white cell count of 15.9 × 10^9^/L (normal range: 5.0–12 × 10^9^/L) with neutrophilia, procalcitonin of 7.6 ng/mL (normal range <0.15 ng/mL), and normal liver and renal function tests. Urinalysis and chest X-ray were normal.

**Figure 1:**
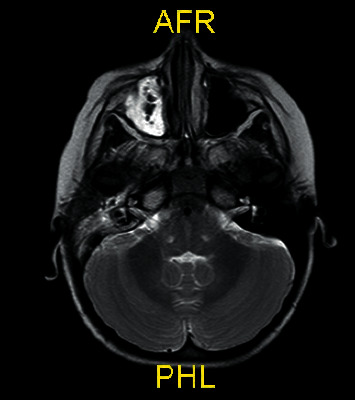
Effusion in right mastoid cells and marked mucosal thickening in the right maxillary sinus.

She was hospitalized, and ceftriaxone was ordered with a daily dose of 100 mg/kg. Her temporal computed tomography showed soft tissue densities causing obliteration in the right maxillary sinus with near-obliteration of right mastoid cellulitis. Her cranial magnetic resonance imaging also showed effusion in right mastoid cells and marked mucosal thickening in the right maxillary sinus (Figure 1). Brain parenchymal structures were normal. Based on these findings, the ear-nose-throat division suggested follow-up with parenteral antibiotics. Lumbar puncture was performed; cerebrospinal fluid (CSF) opening pressure was normal, and the appearance was clear. CSF microscopy examination, Gram staining, and acid-resistant bacillus staining were all clear for cells and organisms. CSF biochemical examinations revealed a CSF glucose of 68 mg/dL (with blood glucose: 85 mg/dL) and CSF total protein of 10 mg/dl. The CSF sample was sent for a multiplex PCR examination. Vancomycin and acyclovir were added to her treatment. There was no growth in the patient's blood, urine, CSF, or throat cultures.

On the sixth day of antibiotic treatment, the child's mother reported that she had a headache. The patient wanted to lie on her right side, her right eye could not look to the right, and she complained of headache and earache. In her repeated contrast cranial CT imaging, bilateral mucosal thickening was observed in the bilateral maxillary and ethmoid sinuses, without aeration in the right mastoid cells and the middle ear was observed. The findings were consistent with OM and mastoiditis.

**Figure 2:**
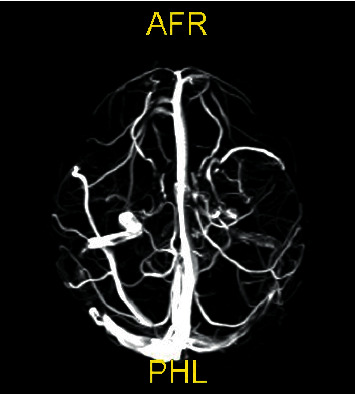
Hyperintense acute stage thrombus in the right transverse sinus and sigmoid sinus.

In her MR angiography study, a compatible appearance with the hyperintense acute stage thrombus was observed in T1-weighted images in the right transverse sinus and sigmoid sinus (Figure 2). There was no pathological signal in the nervus abducens trace. She underwent urgent right mastoidectomy and right ventilation tube insertion into the ear. The Pediatric Hematology Department recommended starting enoxaparin (2 mg/kg/day) after sending a thrombosis panel study (factor II, factor V, and PAI-1 mutation were normal; MHTFR 677 mutation and MTHFR 1298 mutation were heterozygotes, protein S and protein C levels were normal; homocysteine and lipoprotein A were also in expected values).

Meanwhile, her detailed ophthalmological examination revealed right papilledema, and diazoxide therapy was started. During her follow-up, her fever decreased; her CSF multiplex PCR was negative for herpes simplex virus type 1 and 2, as well as *Streptococcus pneumoniae*, enterovirus, and *Neisseria meningitidis* PCR results, but the *H. influenza* result was positive. Based on these findings, acyclovir treatment was stopped, and vancomycin and ceftriaxone continued for four weeks.

We further analyzed the CSF sample using the following procedures; *Haemophilus influenzae* DNA isolated from the patient's cerebrospinal fluid with meningitis was sent to the General Directorate of Public Health, National Respiratory Pathogens Reference Laboratory (USYPRL), for molecular serotyping. The diagnosis of *H. influenzae* was confirmed by applying the type-specific real-time PCR method targeting the HPD genes encoding protein D of *H. influenzae* in USYPRL. Then, serotyping was performed by real-time PCR using serotype-specific primary-probes targeting a, b, c, d, e, and f capsular serotypes of *Haemophilus influenza* [5, 6]. No positivity was observed with serotype-specific primers. Thus, the DNA obtained from the case of CSF belonged to nontypeable *H. influenzae*.

On the 14th day of heparin treatment, her cranial angiography was repeated, and close to complete resolution of the thrombus was observed (Figure 2). She was discharged with heparin for three months, and her control cranial MR angiography was normal.

## 3. Discussion

Here, we report a rare case of meningitis complicated by transverse sinus and sigmoid sinus thrombosis, initially presented as acute OM and mastoiditis. This case demonstrates that NTHi should be considered a potential pathogen that can cause meningitis even in previously healthy children. After introducing universal Hib vaccination, there was an overall decrease in invasive *H. influenzae* infections due to the near elimination of the disease by type B strains [4].

Most invasive *H. influenzae* infections in countries where the *H. influenzae* type B vaccines are used are caused by nontypeable strains [4]. Some but not all studies show an increasing incidence of invasive infections, including bacteremia and meningitis, due to nontypeable *H. influenzae* [4]. NTHi is part of the normal flora in children's upper respiratory tract and commonly causes local respiratory tract diseases, including OM, sinusitis, conjunctivitis, and bronchitis. In our patient, the initial clinical presentation started as acute OM treated with an oral antibiotic. Two weeks later, a second attack presented as purulent OM with perforated tympanic membrane mastoiditis.

It is well known that nontypeable *H. influenzae* is the second most common bacterial cause of OM after *Streptococcus pneumoniae*, causing 25–35% of acute OM [6]. It is not unique, but features associated with nontypeable *H. influenzae* OM include a history of recurrent episodes, treatment failure, concomitant conjunctivitis, previous amoxicillin treatment, bilateral OM, and acute OM within two weeks of completing a course of any antibiotics [4]. As in our patient, a second OM attack was observed two weeks after initial AOM treatment.

After introducing pneumococcal conjugate vaccines (PCV), a significant increase in the proportion of acute OM caused by nontypeable *H. influenzae* in children failing initial antimicrobial therapy or with recurrent episodes was observed [4]. Perhaps, this has also been happening in Turkey.

PCV7 was included in the National Immunization Program (NIP) of Turkey in November 2008 and was used until late 2011, with PCV13 replacing PCV7 in the NIP in April 2011 [7]. After introducing PCV13 into the Turkish NIP, the incidence of AOM has decreased in children less than five years old. Still, the incidence of recurrent AOM has increased in all pediatric age groups [8].

A potential complication of OM is that suppurative fluid from the middle ear can extend to the adjacent anatomical locations, resulting in tympanic membrane (TM) perforation, mastoiditis, labyrinthitis, petrositis, meningitis, brain abscess, hearing loss, and lateral and cavernous sinus thrombosis [9].

In our patient, we initially observed tympanic membrane perforation and mastoiditis two weeks later during an initial AOM attack resulted in central nervous system invasion leading to meningitis. NTHi meningitis in children is rare, in contrast to the hematogenic spread of Hib-meningitis, and infections with NTHi mostly spread to the meninges directly from a local focus [10]. NTHi is often associated with underlying conditions, such as head trauma, neurosurgical implants and devices, compromised immune system, and neonates with maternal genital tract colonization [11].

In our patient, the infection focus was mostly OM, leading to mastoiditis that eventually spreads to brain structures, ultimately causing meningitis complicated with sinus thrombosis. The literature suggests that cases in previously well children with no known risk factors are less common, though these do also occur. In our case, the child had no predisposing factors identified, and immunological testing did not reveal any underlying impairment. The thrombotic disease is uncommon in children, and cerebral venous thrombosis is rare. Jackson et al. estimated an annual incidence of 0.67 cases/100,000 children [12]. Cerebral sinovenous thrombosis (CSVT) has been reported in association with a variety of clinical conditions. Infection and dehydration are the most frequent antecedent illnesses to pediatric CSVT in previously healthy children and those with chronic disease [12]. The most common acquired conditions leading to CSVT in children are OM and mastoiditis, and then meningitis [12], as in our patient. Cerebral sinovenous thrombosis, more commonly involving the lateral venous sinuses (i.e., transverse and sigmoid), is a known potential intracranial complication of OM, both acute and chronic, and can occur in up to 2% of OM cases, often in association with mastoiditis [12].

CSVT has also been reported with meningitis caused by group B *streptococcus*, *Mycobacterium tuberculosis*, *Neisseria meningitidis*, *Fusobacterium necrophorum*, and *Listeria monocytogenes* in children and *Streptococcus pneumoniae*, Aspergillus, alpha-hemolytic *Streptococcus*, *Borrelia burgdorferi, Proteus vulgaris*, and in adults [12–21]. To our knowledge, CSVT has not been reported in the literature for NTHi. Still, previously, a 12-month-old immunocompetent female developed meningitis caused by *Haemophilus influenzae* type F complicated bilateral subdural empyemas, central venous thrombosis, and bilateral sensorineural hearing loss requiring cochlear implants [22]. Treatment of otogenic CVST includes appropriate antibiotic therapy for 4–6 weeks, and operative management ranges from myringotomy with ventilation tube placement alone to myringotomy, tube placement, and mastoidectomy. Besides, anticoagulation treatment is also recommended with low-molecular-weight heparin for at least three months [12]. This case report highlights the importance of NTHi, especially in immunocompetent children with recurrent OM that might be complicated by unwanted rare clinical conditions that have a higher mortality and morbidity rate.

## Data Availability

The data used to support the findings of this study are available from the corresponding author upon request.
